# 3-(4-Bromo­phenyl­sulfin­yl)-2,5-dimethyl-1-benzofuran

**DOI:** 10.1107/S1600536812002656

**Published:** 2012-02-04

**Authors:** Hong Dae Choi, Pil Ja Seo, Uk Lee

**Affiliations:** a Department of Chemistry, Dongeui University, San 24 Kaya-dong Busanjin-gu, Busan 614-714, Republic of Korea; b Department of Chemistry, Pukyong National University, 599-1 Daeyeon 3-dong Nam-gu, Busan 608-737, Republic of Korea

## Abstract

In the title compound, C_16_H_13_BrO_2_S, the 4-bromo­phenyl ring makes a dihedral angle of 87.87 (6)° with the mean plane of the benzofuran fragment. In the crystal, mol­ecules are linked by a weak π–π inter­action between the 4-bromo­phenyl rings [centroid-to-centroid distance = 3.907 (3) Å, inter­planar distance = 3.528 (3) Å and slippage = 1.679 (3) Å].

## Related literature

For the pharmacological activity of benzofuran compounds, see: Aslam *et al.* (2009 ▶); Galal *et al.* (2009 ▶); Khan *et al.* (2005 ▶). For natural products with benzofuran rings, see: Akgul & Anil (2003 ▶); Soekamto *et al.* (2003 ▶). For the crystal structures of related compounds, see: Choi *et al.* (2010*a* ▶,*b* ▶).
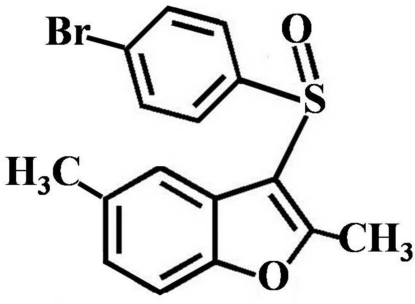


## Experimental

### 

#### Crystal data

C_16_H_13_BrO_2_S
*M* *_r_* = 349.23
Triclinic, 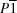

*a* = 6.4145 (3) Å
*b* = 10.0266 (5) Å
*c* = 11.7639 (6) Å
α = 101.606 (3)°
β = 92.240 (2)°
γ = 103.932 (2)°
*V* = 716.28 (6) Å^3^
*Z* = 2
Mo *K*α radiation
μ = 3.01 mm^−1^
*T* = 173 K
0.30 × 0.23 × 0.20 mm

#### Data collection

Bruker SMART APEXII CCD diffractometer
Absorption correction: multi-scan (*SADABS*; Bruker, 2009 ▶) *T*_min_ = 0.518, *T*_max_ = 0.746
12848 measured reflections
3507 independent reflections
3048 reflections with *I* > 2σ(*I*)
*R*_int_ = 0.044

#### Refinement

*R*[*F* ^2^ > 2σ(*F* ^2^)] = 0.033
*wR*(*F* ^2^) = 0.087
*S* = 1.04
3507 reflections
183 parameters
H-atom parameters constrained
Δρ_max_ = 0.57 e Å^−3^
Δρ_min_ = −0.59 e Å^−3^

### 

Data collection: *APEX2* (Bruker, 2009 ▶); cell refinement: *SAINT* (Bruker, 2009 ▶); data reduction: *SAINT*; program(s) used to solve structure: *SHELXS97* (Sheldrick, 2008 ▶); program(s) used to refine structure: *SHELXL97* (Sheldrick, 2008 ▶); molecular graphics: *ORTEP-3* (Farrugia, 1997 ▶) and *DIAMOND* (Brandenburg, 1998 ▶); software used to prepare material for publication: *SHELXL97*.

## Supplementary Material

Crystal structure: contains datablock(s) global, I. DOI: 10.1107/S1600536812002656/kp2381sup1.cif

Structure factors: contains datablock(s) I. DOI: 10.1107/S1600536812002656/kp2381Isup2.hkl

Supplementary material file. DOI: 10.1107/S1600536812002656/kp2381Isup3.cml

Additional supplementary materials:  crystallographic information; 3D view; checkCIF report

## Figures and Tables

**Fig. 1.**
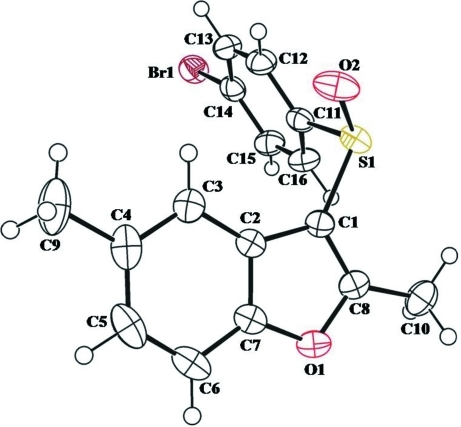
The molecular structure of the title compound with the atom numbering scheme. Displacement ellipsoids are drawn at the 50% probability level. H atoms are presented as small spheres of arbitrary radius.

**Fig. 2.**
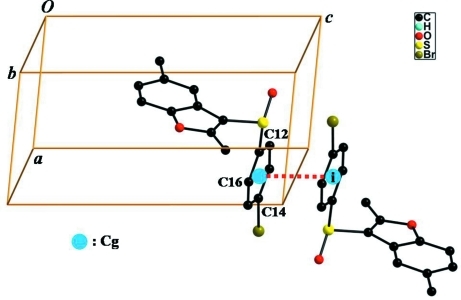
A view of the π–π interactions (dotted lines) in the crystal structure of the title compound. All H atoms were omitted for clarity. [Symmetry codes: (i) -x + 2, -y + 1, -z + 2].

## supplementary crystallographic information

### Comment

Many compounds involving a benzofuran ring have drawn much attention owing to their valuable biological properties such as antibacterial, antifungal, antitumor, and antiviral activities (Aslam *et al.*, 2009; Galal *et al.*, 2009; Khan *et al.*, 2005). These benzofuran derivatives occur in a wide range of natural products (Akgul & Anil, 2003; Soekamto *et al.*, 2003). As a part of our continuing study of 2,5-dimethyl-1-benzofuran derivatives containing either 3-(4-fluorophenylsulfinyl) (Choi *et al.*, 2010*a*) or 3-(4-chlorophenylsufinyl) (Choi *et al.*, 2010*b*) substituents, we report herein the crystal structure of the title compound.

In the title molecule (Fig. 1), the benzofuran unit is planar with the mean deviation of 0.006 (2) Å from the least-squares plane defined by the nine constituent atoms. The dihedral angle between the 4-bromophenyl ring and the mean plane of the benzofuran fragment is 87.87 (6)°. The crystal packing (Fig. 2) is further stabilised by a weak π–π interaction between the 4-bromophenyl rings of adjacent molecules, with a Cg···Cg^i^ distance of 3.907 (3) Å and an interplanar distance of 3.528 (3) Å resulting in a slippage of 1.679 (3) Å (Cg is the centroid of the C11–C16 4-bromophenyl ring).

### Experimental

3-Chloroperoxybenzoic acid 77% (269 mg, 1.2 mmol) was added in small portions to a stirred solution of 3-(4-bromophenylsulfanyl)-2,5-dimethyl-1-benzofuran (366 mg, 1.1 mmol) in dichloromethane (40 ml) at 273 K. After being stirred at room temperature for 5 h, the mixture was washed with saturated sodium bicarbonate solution and the organic layer was separated, dried over magnesium sulfate, filtered and concentrated at reduced pressure. The residue was purified by column chromatography (hexane–ethyl acetate, 2:1 v/v) to afford the title compound as a colourless solid [yield 72%, m.p. 434–435 K; *R*_f_ = 0.49 (hexane–ethyl acetate, 2:1 v/v)]. Single crystals suitable for X-ray diffraction were prepared by slow evaporation of a solution of the title compound in ethyl acetate at room temperature.

### Refinement

All H atoms were positioned geometrically and refined using a riding model, with C—H = 0.95 Å for aryl and 0.98 Å for methyl H atoms. *U*_iso_(H) = 1.2*U*_eq_(C) for aryl and 1.5*U*_eq_(C)for methyl H atoms.

### Crystal data

**Table:**

C_16_H_13_BrO_2_S	*Z* = 2
*M**_r_* = 349.23	*F*(000) = 352
Triclinic, *P*1	*D*_x_ = 1.619 Mg m^−^^3^
Hall symbol: -P 1	Mo *K*α radiation, λ = 0.71073 Å
*a* = 6.4145 (3) Å	Cell parameters from 7068 reflections
*b* = 10.0266 (5) Å	θ = 2.5–28.2°
*c* = 11.7639 (6) Å	µ = 3.01 mm^−^^1^
α = 101.606 (3)°	*T* = 173 K
β = 92.240 (2)°	Block, colourless
γ = 103.932 (2)°	0.30 × 0.23 × 0.20 mm
*V* = 716.28 (6) Å^3^	

### Data collection

**Table:**

Bruker SMART APEXII CCD diffractometer	3507 independent reflections
Radiation source: rotating anode	3048 reflections with *I* > 2σ(*I*)
Graphite multilayer monochromator	*R*_int_ = 0.044
Detector resolution: 10.0 pixels mm^-1^	θ_max_ = 28.3°, θ_min_ = 1.8°
φ and ω scans	*h* = −8→8
Absorption correction: multi-scan (*SADABS*; Bruker, 2009)	*k* = −13→13
*T*_min_ = 0.518, *T*_max_ = 0.746	*l* = −15→15
12848 measured reflections	

### Refinement

**Table:**

Refinement on *F*^2^	Primary atom site location: structure-invariant direct methods
Least-squares matrix: full	Secondary atom site location: difference Fourier map
*R*[*F*^2^ > 2σ(*F*^2^)] = 0.033	Hydrogen site location: difference Fourier map
*wR*(*F*^2^) = 0.087	H-atom parameters constrained
*S* = 1.04	*w* = 1/[σ^2^(*F*_o_^2^) + (0.0431*P*)^2^ + 0.3244*P*] where *P* = (*F*_o_^2^ + 2*F*_c_^2^)/3
3507 reflections	(Δ/σ)_max_ = 0.001
183 parameters	Δρ_max_ = 0.57 e Å^−^^3^
0 restraints	Δρ_min_ = −0.59 e Å^−^^3^

### Special details

**Table:**

Geometry. All esds (except the esd in the dihedral angle between two l.s. planes) are estimated using the full covariance matrix. The cell esds are taken into account individually in the estimation of esds in distances, angles and torsion angles; correlations between esds in cell parameters are only used when they are defined by crystal symmetry. An approximate (isotropic) treatment of cell esds is used for estimating esds involving l.s. planes.
Refinement. Refinement of F^2^ against ALL reflections. The weighted R-factor wR and goodness of fit S are based on F^2^, conventional R-factors R are based on F, with F set to zero for negative F^2^. The threshold expression of F^2^ > 2sigma(F^2^) is used only for calculating R-factors(gt) etc. and is not relevant to the choice of reflections for refinement. R-factors based on F^2^ are statistically about twice as large as those based on F, and R- factors based on ALL data will be even larger.

### Fractional atomic coordinates and isotropic or equivalent isotropic displacement parameters (Å^2^)

**Table:**

	*x*	*y*	*z*	*U*_iso_*/*U*_eq_	
Br1	1.23629 (4)	0.91785 (2)	0.915580 (19)	0.04170 (10)	
S1	0.69071 (9)	0.27380 (6)	0.84528 (4)	0.03194 (13)	
O1	0.8013 (2)	0.10195 (16)	0.53135 (13)	0.0336 (3)	
O2	0.4646 (3)	0.2757 (2)	0.86938 (15)	0.0462 (4)	
C1	0.6970 (3)	0.2139 (2)	0.69530 (17)	0.0274 (4)	
C2	0.5786 (3)	0.2405 (2)	0.59942 (17)	0.0265 (4)	
C3	0.4234 (4)	0.3139 (2)	0.5861 (2)	0.0332 (5)	
H3	0.3725	0.3633	0.6524	0.040*	
C4	0.3442 (4)	0.3134 (3)	0.4742 (2)	0.0396 (5)	
C5	0.4239 (4)	0.2411 (3)	0.3781 (2)	0.0453 (6)	
H5	0.3709	0.2433	0.3022	0.054*	
C6	0.5762 (4)	0.1665 (3)	0.3889 (2)	0.0410 (5)	
H6	0.6276	0.1169	0.3229	0.049*	
C7	0.6492 (3)	0.1686 (2)	0.50126 (18)	0.0303 (4)	
C8	0.8253 (3)	0.1306 (2)	0.65040 (18)	0.0298 (4)	
C9	0.1740 (4)	0.3903 (3)	0.4573 (3)	0.0570 (8)	
H9A	0.0339	0.3220	0.4338	0.086*	
H9B	0.2114	0.4437	0.3966	0.086*	
H9C	0.1660	0.4550	0.5305	0.086*	
C10	0.9846 (4)	0.0710 (3)	0.7040 (2)	0.0406 (5)	
H10A	1.1308	0.1223	0.6938	0.061*	
H10B	0.9612	−0.0286	0.6664	0.061*	
H10C	0.9671	0.0797	0.7874	0.061*	
C11	0.8359 (3)	0.4538 (2)	0.85581 (16)	0.0277 (4)	
C12	0.7417 (4)	0.5610 (2)	0.89976 (19)	0.0334 (5)	
H12	0.5963	0.5394	0.9183	0.040*	
C13	0.8589 (4)	0.7004 (2)	0.91705 (19)	0.0354 (5)	
H13	0.7951	0.7747	0.9475	0.042*	
C14	1.0693 (4)	0.7290 (2)	0.88928 (17)	0.0304 (4)	
C15	1.1664 (4)	0.6220 (2)	0.84572 (19)	0.0344 (5)	
H15	1.3117	0.6438	0.8269	0.041*	
C16	1.0498 (4)	0.4837 (2)	0.83008 (19)	0.0342 (5)	
H16	1.1152	0.4094	0.8019	0.041*	

### Atomic displacement parameters (Å^2^)

**Table:**

	*U*^11^	*U*^22^	*U*^33^	*U*^12^	*U*^13^	*U*^23^
Br1	0.05053 (18)	0.03378 (14)	0.03637 (14)	0.00588 (11)	0.00285 (10)	0.00355 (9)
S1	0.0324 (3)	0.0376 (3)	0.0233 (2)	0.0047 (2)	0.0038 (2)	0.0056 (2)
O1	0.0309 (8)	0.0348 (8)	0.0329 (8)	0.0088 (7)	0.0073 (6)	0.0011 (6)
O2	0.0323 (9)	0.0570 (11)	0.0400 (9)	−0.0004 (8)	0.0154 (7)	0.0009 (8)
C1	0.0257 (10)	0.0285 (10)	0.0262 (9)	0.0053 (8)	0.0017 (8)	0.0039 (8)
C2	0.0227 (10)	0.0271 (10)	0.0267 (9)	0.0010 (8)	0.0024 (7)	0.0051 (8)
C3	0.0261 (11)	0.0345 (11)	0.0393 (12)	0.0057 (9)	0.0036 (9)	0.0107 (9)
C4	0.0250 (11)	0.0403 (12)	0.0530 (14)	−0.0023 (9)	−0.0041 (10)	0.0235 (11)
C5	0.0367 (13)	0.0582 (16)	0.0352 (12)	−0.0070 (11)	−0.0066 (10)	0.0214 (11)
C6	0.0392 (13)	0.0504 (14)	0.0256 (10)	−0.0011 (11)	0.0024 (9)	0.0056 (9)
C7	0.0263 (11)	0.0316 (10)	0.0284 (10)	0.0005 (8)	0.0028 (8)	0.0042 (8)
C8	0.0266 (11)	0.0262 (10)	0.0334 (10)	0.0036 (8)	0.0022 (8)	0.0032 (8)
C9	0.0308 (14)	0.0602 (17)	0.086 (2)	0.0058 (12)	−0.0083 (13)	0.0402 (16)
C10	0.0327 (13)	0.0368 (12)	0.0536 (14)	0.0123 (10)	0.0005 (11)	0.0092 (10)
C11	0.0273 (10)	0.0342 (10)	0.0201 (9)	0.0073 (8)	0.0007 (7)	0.0029 (7)
C12	0.0263 (11)	0.0439 (12)	0.0320 (11)	0.0135 (9)	0.0066 (8)	0.0065 (9)
C13	0.0388 (13)	0.0388 (12)	0.0316 (10)	0.0182 (10)	0.0056 (9)	0.0038 (9)
C14	0.0350 (12)	0.0308 (10)	0.0224 (9)	0.0061 (9)	−0.0023 (8)	0.0026 (8)
C15	0.0263 (11)	0.0395 (12)	0.0358 (11)	0.0090 (9)	0.0052 (9)	0.0035 (9)
C16	0.0303 (11)	0.0372 (12)	0.0344 (11)	0.0117 (9)	0.0079 (9)	0.0011 (9)

### Geometric parameters (Å, º)

**Table:**

Br1—C14	1.895 (2)	C8—C10	1.482 (3)
S1—O2	1.4927 (18)	C9—H9A	0.9800
S1—C1	1.751 (2)	C9—H9B	0.9800
S1—C11	1.798 (2)	C9—H9C	0.9800
O1—C8	1.367 (3)	C10—H10A	0.9800
O1—C7	1.381 (3)	C10—H10B	0.9800
C1—C8	1.357 (3)	C10—H10C	0.9800
C1—C2	1.439 (3)	C11—C12	1.379 (3)
C2—C7	1.386 (3)	C11—C16	1.391 (3)
C2—C3	1.395 (3)	C12—C13	1.388 (3)
C3—C4	1.391 (3)	C12—H12	0.9500
C3—H3	0.9500	C13—C14	1.377 (3)
C4—C5	1.399 (4)	C13—H13	0.9500
C4—C9	1.508 (3)	C14—C15	1.389 (3)
C5—C6	1.382 (4)	C15—C16	1.379 (3)
C5—H5	0.9500	C15—H15	0.9500
C6—C7	1.380 (3)	C16—H16	0.9500
C6—H6	0.9500		
			
O2—S1—C1	108.74 (10)	C4—C9—H9B	109.5
O2—S1—C11	106.52 (10)	H9A—C9—H9B	109.5
C1—S1—C11	97.81 (10)	C4—C9—H9C	109.5
C8—O1—C7	106.09 (16)	H9A—C9—H9C	109.5
C8—C1—C2	107.82 (18)	H9B—C9—H9C	109.5
C8—C1—S1	122.92 (16)	C8—C10—H10A	109.5
C2—C1—S1	129.26 (16)	C8—C10—H10B	109.5
C7—C2—C3	119.4 (2)	H10A—C10—H10B	109.5
C7—C2—C1	104.22 (18)	C8—C10—H10C	109.5
C3—C2—C1	136.4 (2)	H10A—C10—H10C	109.5
C4—C3—C2	118.9 (2)	H10B—C10—H10C	109.5
C4—C3—H3	120.6	C12—C11—C16	120.6 (2)
C2—C3—H3	120.6	C12—C11—S1	119.81 (17)
C3—C4—C5	119.4 (2)	C16—C11—S1	119.33 (16)
C3—C4—C9	120.0 (3)	C11—C12—C13	120.2 (2)
C5—C4—C9	120.6 (2)	C11—C12—H12	119.9
C6—C5—C4	122.9 (2)	C13—C12—H12	119.9
C6—C5—H5	118.5	C14—C13—C12	118.8 (2)
C4—C5—H5	118.5	C14—C13—H13	120.6
C7—C6—C5	115.9 (2)	C12—C13—H13	120.6
C7—C6—H6	122.0	C13—C14—C15	121.5 (2)
C5—C6—H6	122.0	C13—C14—Br1	119.97 (16)
C6—C7—O1	125.3 (2)	C15—C14—Br1	118.46 (17)
C6—C7—C2	123.5 (2)	C16—C15—C14	119.3 (2)
O1—C7—C2	111.16 (18)	C16—C15—H15	120.3
C1—C8—O1	110.71 (18)	C14—C15—H15	120.3
C1—C8—C10	133.1 (2)	C15—C16—C11	119.6 (2)
O1—C8—C10	116.15 (19)	C15—C16—H16	120.2
C4—C9—H9A	109.5	C11—C16—H16	120.2
			
O2—S1—C1—C8	−143.25 (19)	C1—C2—C7—O1	−0.3 (2)
C11—S1—C1—C8	106.29 (19)	C2—C1—C8—O1	0.8 (2)
O2—S1—C1—C2	37.3 (2)	S1—C1—C8—O1	−178.83 (14)
C11—S1—C1—C2	−73.2 (2)	C2—C1—C8—C10	178.7 (2)
C8—C1—C2—C7	−0.3 (2)	S1—C1—C8—C10	−0.9 (4)
S1—C1—C2—C7	179.30 (17)	C7—O1—C8—C1	−1.0 (2)
C8—C1—C2—C3	178.9 (2)	C7—O1—C8—C10	−179.27 (19)
S1—C1—C2—C3	−1.5 (4)	O2—S1—C11—C12	10.2 (2)
C7—C2—C3—C4	−0.3 (3)	C1—S1—C11—C12	122.51 (17)
C1—C2—C3—C4	−179.4 (2)	O2—S1—C11—C16	−175.97 (17)
C2—C3—C4—C5	−0.8 (3)	C1—S1—C11—C16	−63.70 (18)
C2—C3—C4—C9	179.1 (2)	C16—C11—C12—C13	1.1 (3)
C3—C4—C5—C6	1.4 (4)	S1—C11—C12—C13	174.76 (16)
C9—C4—C5—C6	−178.5 (2)	C11—C12—C13—C14	0.2 (3)
C4—C5—C6—C7	−0.8 (4)	C12—C13—C14—C15	−0.6 (3)
C5—C6—C7—O1	−179.6 (2)	C12—C13—C14—Br1	−178.34 (16)
C5—C6—C7—C2	−0.4 (4)	C13—C14—C15—C16	−0.1 (3)
C8—O1—C7—C6	−179.9 (2)	Br1—C14—C15—C16	177.61 (17)
C8—O1—C7—C2	0.8 (2)	C14—C15—C16—C11	1.3 (3)
C3—C2—C7—C6	0.9 (3)	C12—C11—C16—C15	−1.8 (3)
C1—C2—C7—C6	−179.7 (2)	S1—C11—C16—C15	−175.56 (17)
C3—C2—C7—O1	−179.69 (18)		
